# Shuni Virus Replicates at the Maternal-Fetal Interface of the Ovine and Human Placenta

**DOI:** 10.3390/pathogens10010017

**Published:** 2020-12-29

**Authors:** Judith Oymans, Lucien van Keulen, Guus M. Vermeulen, Paul J. Wichgers Schreur, Jeroen Kortekaas

**Affiliations:** 1 Department of Virology, Wageningen Bioveterinary Research, Houtribweg 39, 8221 RA Lelystad, The Netherlands; j.oymans@uu.nl (J.O.); lucien.vankeulen@wur.nl (L.v.K.); paul.wichgersschreur@wur.nl (P.J.W.S.); 2 Laboratory of Virology, Wageningen University & Research, P.O. Box 16, 6700 AA Wageningen, The Netherlands; 3 Department of Gynaecology, Isala Hospital, 8025 AB Zwolle, The Netherlands; g.vermeulen@isala.nl

**Keywords:** Shuni virus, vertical transmission, pregnant ewes, placenta, explants, zoonosis

## Abstract

Shuni virus (SHUV) is a neglected teratogenic and neurotropic orthobunyavirus that was discovered in the 1960s in Nigeria and was subsequently detected in South Africa, Zimbabwe, and Israel. The virus was isolated from field-collected biting midges and mosquitoes and shown to disseminate efficiently in laboratory-reared biting midges, suggesting that members of the families *Culicidae* and *Ceratopogonidae* may function as vectors. SHUV infections have been associated with severe neurological disease in horses, a variety of wildlife species, and domesticated ruminants. SHUV infection of ruminants is additionally associated with abortion, stillbirth, and congenital malformations. The detection of antibodies in human sera also suggests that the virus may have zoonotic potential. To understand how SHUV crosses the ruminant placenta, we here infected pregnant ewes and subsequently performed detailed clinical- and histopathological examination of placental tissue. We found that SHUV targets both maternal epithelial cells and fetal trophoblasts, that together form the maternal-fetal interface of the ovine placenta. Experiments with human placental explants, furthermore, revealed replication of SHUV in syncytiotrophoblasts, which are generally highly resistant to virus infections. Our findings provide novel insights into vertical transmission of SHUV in sheep and call for research on the potential risk of SHUV infection during human pregnancies.

## 1. Introduction

Shuni virus (SHUV) is an orthobunyavirus of the family *Peribunyaviridae* that was first discovered in 1966 in Ibadan, Nigeria, where it was isolated from the serum of an adult cow and a febrile child [1,2]. In subsequent years, SHUV was isolated from cattle, goats, and horses in South Africa and Zimbabwe. In 2014, SHUV emerged in Israel, representing its first detection outside the African continent [3]. SHUV was isolated from various field-collected *Culicoides* biting midges and *Culex theileri* mosquitoes, suggesting that both midges and mosquitoes may act as vectors [4,5]. A recent study has demonstrated efficient dissemination of SHUV in laboratory-reared *Culicoides nubeculosus* and *Culicoides sonorensis* biting midges but not in laboratory-reared *Culex pipiens* and *Aedes aegypti* mosquitoes [6].

SHUV affects a wide variety of vertebrate host species. The virus infects domesticated ruminants, horses, and a wide variety of wildlife species, such as rhinos, crocodiles. and giraffes [7,8,9]. In horses, cattle, and wildlife, SHUV causes severe neurological disease manifesting with tremors and ataxia [7,9,10]. In domesticated ruminants, SHUV is capable of crossing the placental barrier, resulting in congenital malformations, stillbirth, or abortion [11]. Importantly, SHUV-specific antibodies were detected in 5 (3.9%) of 123 veterinarians from South Africa in a serological survey, warranting further research into its zoonotic potential [12].

Congenital malformations, stillbirths, and abortions of ruminant livestock are characteristic outcomes of infections caused by orthobunyaviruses of the Simbu serogroup. The most widespread orthobunyavirus of the Simbu serogroup affecting ruminants is Akabane virus (AKAV), which is endemic to Africa, the Middle East, Asia, and Australia [13,14]. Additional Simbu serogroup orthobunyaviruses of veterinary importance include Schmallenberg virus (SBV), Aino virus, Peaton virus, and Shamonda virus [15,16,17,18]. Examples of orthobunyaviruses pathogenic to humans include La Crosse virus, a major cause of encephalitis in children in the U.S., Oropouche virus, which generally causes a febrile illness, and Ngari virus, associated with hemorrhagic fever [19,20,21]. Orthobunyaviruses infecting humans have not been associated with congenital malformations.

Our understanding of vertical transmission of teratogenic arboviruses remains rudimentary. Improved understanding of the mechanisms involved in vertical transmission of arboviruses in both animals and humans may facilitate prioritization of arbovirus research and the development of countermeasures. To further our understanding of vertical transmission of SHUV, we experimentally inoculated pregnant ewes at one-third of gestation and identified maternal epithelial cells and fetal trophoblasts as target cells of SHUV. Moreover, as SHUV is the only known teratogenic orthobunyavirus with zoonotic potential, we examined the interaction of SHUV with human placental explants. The results of these experiments demonstrate that SHUV replicates efficiently in human syncytiotrophoblasts, the placental cells that form the primary barrier between mother and fetus.

## 2. Materials and Methods 

### 2.1. Viruses and Cells

Culture media and supplements were obtained from Gibco (Thermo Fisher Scientific, Breda, the Netherlands), unless stated otherwise. SHUV strain Iban101007 was obtained from the World Reference Center for Emerging Viruses and Arboviruses (WRCEVA) through the University of Texas Medical Branch. This strain was originally isolated from serum of a cow in a slaughterhouse [1]. The virus was passaged twice in Vero E6 cells before use in the described experiments. Virus titer was determined by end-point dilution assay using Vero E6 cells and subsequent staining with a SHUV-specific antiserum targeting the head domain of SHUV glycoprotein C (Gc) (SHUV-Gc_head_ [22]) and calculated using the Spearman-Kärber algorithm [23,24].

Vero E6 cells, obtained from the American Type Culture Collection (ATCC), were maintained in minimal essential medium (MEM) supplemented with 5% fetal bovine serum (FBS), and 1% antibiotic/antimycotic (a/a), 1% glutamine, and 1% Minimal Essential Medium Non-Essential Amino Acids (MEM NEAA). Human umbilical vein endothelial (HUVEC) cells (ATCC^®^ CRL-1730™) were maintained in Ham’s F-12K (Kaighn’s) medium supplemented with 10% FBS, 0.03 mg/mL Endothelial Cell Growth Supplement (ECGS), and 0.002% heparin. Human trophoblasts cells (HTR-8) cells (ATCC^®^ CRL-3271™) were maintained in RPMI medium supplemented with 5% FBS and 1% a/a. All cells were cultured at 37 °C with 5% CO_2_.

To develop immortalized ovine placental cells (OPC), the fetal and maternal parts of a placentome were separated. The maternal part (caruncle) was attached to a sterile cotton and subsequently submerged in 90% DMEM/Ham’s F12 medium supplemented with 200 U/mL collagenase I and 10% Hank’s Salt solution (Biochrom, Berlin, Germany). After 1 h, the tissue was discarded, and the cell suspension was cultured in DMEM/Ham’ F12 medium supplemented with 10% FBS and 1% a/a. After 2 passages, a heterogenous cell suspension was sent to InSCREENeX GmbH, Braunschweig, Germany, to immortalize single cells. Fifteen clones were provided and cultured in DMEM/Ham’s F12 medium supplemented with 10% FBS and 1% a/a. Clone #16 (OPC-16) showed best growth kinetics; therefore, this cell line was selected for the present work.

Titrations were performed by end-point dilution assay on Vero cell monolayers in 96 well plates, followed by immunostaining of SHUV antigen using a rabbit polyclonal antiserum [22]. Titers were determined using the Spearman-Kärber algorithm and expressed as 50% tissue culture infective dose (TCID50)/mL.

### 2.2. Ethics Statement

The pregnant ewe trial was conducted in accordance with the Dutch Law on Animal Experiments (Wet op de Dierproeven, ID number BWBR0003081) and the European regulations on the protection of animals used for scientific purposes (EU directive 2010/63/EU). The procedures were approved by the animal ethics committee of Wageningen Bioveterinary Research (WBVR) and the Dutch Central Authority for Scientific Procedures on Animals (permit number AVD401002017894).

Human placentas were obtained from healthy women after caesarean section. Placentas are regarded as medical waste and, therefore, do not fall under the scientific medical research law of the Netherlands. The experiments did not require approval from an institutional review board. All donors have given written consent, and consent forms are stored in accordance with the Dutch privacy law.

### 2.3. Pregnant Ewe Trial

At a conventional Dutch sheep farm the pregnancies of Texel-Swifter mix-breed ewes were synchronized by progesterone sponge treatment after which natural mating occurred. Ultrasounds were performed to confirm pregnancy after which 4 ewes were transported to WBVR on day 41 of gestation. The ewes were allowed to acclimatize for 7 days and the ewes were confirmed negative for SBV antibodies as this virus is closely related to SHUV and might interfere with the experiment. At day 48 of gestation, the ewes were inoculated intravenously with 10^5.6^ TCID_50_ SHUV in 1 mL culture medium. Animals were closely monitored for clinical signs twice per day. Rectal body temperatures were recorded and EDTA blood samples were collected daily. At 7 days post-inoculation, the ewes were euthanized by intravenous inoculation of 50 mg/kg sodium pentobarbital (Euthasol, ASTgarma, Oudewater, the Netherlands) and subsequent exsanguination of both the ewe and the fetuses. From the ewes, samples were taken from the liver, spleen, and the lymph nodes (ln) that drain the placenta, namely *ln iliaca* and *ln inguinales*. From each placenta, three placentomes were collected. From the fetuses samples were taken from the liver, brain, umbilical cord, and fetal blood. All samples were collected in duplicate. One organ sample was stored at −80 °C for RNA extraction and virus isolation, and one sample was stored in 10% neutral buffered formalin for 48 h for histology and immunohistochemistry (IHC).

Infection of human cell lines: monolayers of Vero E6, HUVEC, HTR-8, or OPC-16 cells, were incubated with SHUV at a multiplicity of infection (MOI) of 0.01 in triplicate. Two hours post-infection, medium was removed, and cells were washed twice with phosphate-buffered saline (PBS) before adding fresh medium. Samples were collected at 0, 2, 24, 48, and 72 hpi. 

For isolation of SHUV from serum, 1 mL of serum was added to 40 mL culture medium in a T150 flask containing a monolayer of 4.5 × 10^6^ Vero E6 cells, seeded 6 h earlier. Isolation of SHUV from placentomes was performed by the same protocol using 1 mL of a 1% placentome suspension. Inocula were maintained with the cells until cytopathogenic effect was observed at 4 days post-infection (dpi), followed by collection of supernatant samples. Titers in supernatant samples were determined by end-point dilution assay as described above.

### 2.4. Experimental Infection of Human Term Placentas

Healthy human term placentas were obtained after caesarean section from the Isala hospital in Zwolle, the Netherlands, and processed into explants as described [25]. Explants were either incubated with 2 × 10^4^ TCID_50_/mL SHUV or medium (mock). The placental explants were maintained at 37 °C and 5% CO_2_ under continuous shaking for maximum perfusion of the explant. At 1 dpi, the medium was removed, and explants washed three times with PBS before fresh medium was added. At 3 dpi, 1 mL fresh medium was added to each well to add fresh nutrients. At 1, 2, 4, and 6 dpi, 4 individual explants were collected in 1 mL Trizol (Invitrogen). On the same days, supernatant samples were collected and pooled for virus titration as described above. Samples were stored at −80 °C until analysis. Furthermore, at each time point, four individual explants were fixed with 10% neutral buffered formalin for IHC. Each experiment was performed in quadruplicate and repeated with two different placentas.

### 2.5. Detection of Viral RNA

RNA extractions of organs from the pregnant ewe trial were performed by homogenizing 0.7 g of tissue in an IKA Ultra Turrax Tube DT-20 containing 7 mL of complete Vero medium. Cell debris was subsequently removed by centrifugation and 200 µL organ suspension or plasma was added to 50 µL Proteinase K (5 µg/mL, Sigma-Aldrich, St. Louis, MO, USA), after which 200 µL AL buffer (Qiagen, Venlo, The Netherlands) supplemented with 2 µL polyadenylic acid A (5 mg/mL, Sigma) was added. After thorough mixing, samples were incubated at 56 °C for 15 min. After lysis, RNA was extracted using the Qiagen RNeasy kit according to the manufacturer’s protocol.

Homogenization of placental explants occurred in Lysing Matric D tubes (MP Biomedicals, Eschwege, Germany) in 1 mL Trizol using the TeSeE™ Precess 24 bead beater for 2 × 23 s at 6500 RPM. RNA was isolated form 350 µL supernatant using the Direct-zol RNA miniprep kit (Zymo Research) according to manufacturer’s protocol.

The Lightcycler RNA Amplification kit HybProbe (Roche, Almere, The Netherlands) was used for reverse-transcriptase quantitative PCR (RT-qPCR) with 5 µL of the RNA, employing the following cycling conditions: 45 °C for 30 min, 95 °C for 5 min, 45 cycles of 5 s at 95 °C, and 35 s at 57 °C, followed by cooling down to 30 °C [25]. Primers and probes, targeting the SHUV S segment, were purchased from Integrated DNA technologies (IDT). Forward primer: GAAGGCCAAGATGGTACT, reverse primer: ACAGGATTTGCTGTGTATTG, and probe: FAM-AGTAAGACGGCACAACCGAGTGTT-BHQ1. 

A SHUV standard for quantification of RNA as TCID_50_ equivalent/mL was made by isolating RNA of our virus stock with a known titer (Figure S1), using the method that has been described earlier for Zika and Wesselsbron virus [26]. 

### 2.6. Histology and Immunohistochemistry

Formalin-embedded tissues were routinely processed into paraffin blocks, cut into 4 μm sections, collected on silane-coated glass slides and dried for at least 48 h at 37 °C. After deparaffinization and rehydration in graded alcohols, sections were stained with hematoxylin-eosin (HE) or immunostained. For immunostaining, endogenous peroxidase was blocked for 30 min in methanol/H_2_O_2_, followed by antigen retrieval by treating the cells with 0.2% trypsin in Tris-buffered saline for 30 min at 37 °C. The SHUV polyclonal antiserum was used at a dilution of 1:250 and incubated for 1 h at 37 °C. Alkaline phosphatase (AP; Vector laboratories, Peterborough, UK)-conjugated anti-rabbit IgG polymer was used as secondary antibody, which was incubated with the cells for 30 min at 37 °C. This was followed by incubation with ImmPACT VectorRed substrate (Vector Laboratories, Peterborough, UK) for 20 min at room temperature. Sections were briefly counterstained with hematoxylin, dehydrated, and mounted permanently.

For the detection of cytokeratin 19, a marker of human trophoblasts, epitope retrieval was performed by 15 min of autoclaving at 121 °C in citrate buffer of pH 6 (Antigen unmasking solution, Vector Laboratories), followed by incubation for 1 h at 37 °C with a rabbit monoclonal antibody to cytokeratin 19 (Abcam, Cambridge, UK) at a dilution of 1:400. The next steps were performed as described in the previous paragraph.

For detection of SHUV and cytokeratin 19 by immunofluorescence, a two-step immunostaining was performed to avoid interference between the different procedures. First, sections were incubated with the SHUV antiserum, then autoclaved for 15 min at 121 °C in citrate pH 6 to destroy the antibodies/enzymes of the first staining and to retrieve the epitopes for the second immunostaining for cytokeratin 19. Anti-rabbit horseradish peroxidase-conjugated polymer was used as secondary antibody (Invitrogen, Carlsbad, CA, USA) followed by incubation with Alexa Fluor 488 or 546 tyramide reagent (Invitrogen) and sections were mounted in antifading mounting medium containing DAPI (Vector laboratories). 

Sections were photographed with an Olympus BX51 (fluorescence) microscope equipped with Cell D/Cell Sense software and a high-resolution digital camera (Olympus, Leiderdorp, The Netherlands). Monochromatic digital photographs for immunofluorescence were false colored in green for the Alex Fluor 488 dye and in red for the Alexa Fluor 546 dye.

## 3. Results and Discussion

### 3.1. Vertical Transmission of SHUV in Pregnant Ewes

**Figure 1:**
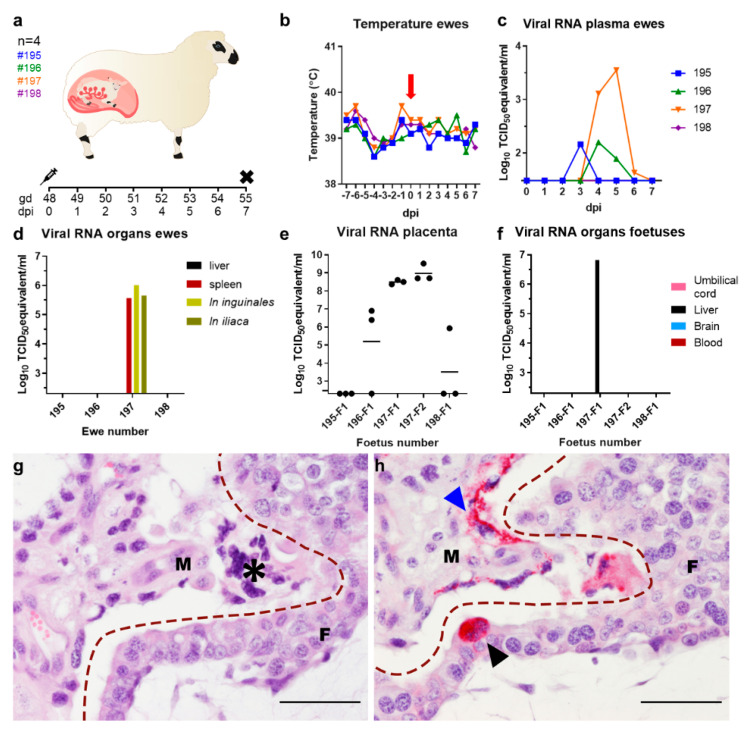
Clinical and laboratory findings following infection of pregnant ewes with Shuni virus (SHUV). (**a**) Experimental set-up of the pregnant ewe trial. Ewes were inoculated at gestation day (gd) 48. EDTA blood samples were collected and rectal temperatures measured daily. At 7 days post-infection (dpi), the ewes were euthanized and tissue samples collected. (**b**) Rectal temperatures. The moment of inoculation is depicted by the red arrow. (**c**) Viral RNA in plasma of the ewes and (**d**) viral RNA in maternal organs. Ln: lymph node (**e**) Viral RNA detected in three placentomes per placenta. Horizontal bars represent means. (**f**) Viral RNA detected in fetal organs. (**g**) Hematoxylin-eosin (HE) staining of a placentome from ewe #197, showing the boundary between a maternal villus (M) and fetal villus (F). Notice the syncytial cell in the maternal epithelium (asterisk). (**h**) Serial section showing the same area staining for SHUV antigen (visualized by VectorRed). SHUV-positive maternal epithelium is indicted by a blue arrowhead. A single SHUV-positive binucleate trophoblast is indicated by a black arrowhead. Dashed lines mark the placental barrier separating maternal (M) and fetal (F) villi. Bars: 50 µm.

To study the interaction of SHUV with the ovine placenta early after exposure, 4 pregnant ewes were inoculated intravenously with a dose of 10^5.6^ TCID_50_ at 48 days of gestation (Figure 1a). No increases in rectal temperatures were measured after inoculation in any of the ewes (Figure 1b). The duration and levels of viremia varied significantly among animals. No viremia was measured in sheep number #198, whereas, in sheep #195 and #196, low levels of viral RNA were detected. In animal #197, higher RNA levels were measured (Figure 1c).

When ewes were necropsied at 7 dpi, all fetuses were alive without visible pathological changes. No abnormalities were noted to the organs of both ewes and fetuses. Analysis by PCR revealed high levels of viral RNA in the spleen and lymph nodes of one ewe, #197 (Figure 1d). No viral RNA was detected in the liver of this animal. High levels of viral RNA were also detected in all 3 of the tested placentomes from each placenta (n = 2) of ewe #197 (Figure 1e). Low amounts of viral RNA were detected in 2 out of 3 placentomes from the single fetus of ewe #196. In the other two animals, no evidence of virus replication was observed. Virus was isolated from plasma collected on day 5 from ewe #197 and from one placentome of the placenta of fetus F2. Viral RNA was detected in the liver of one of the two fetuses of ewe #197 (Figure 1f), confirming that SHUV crossed the placental barrier. 

In order to identify the primary target cells of SHUV in the ovine placenta, tissue sections were analyzed with IHC. Whereas no lesions were detected in the studied placentomes (Figure 1g), small foci of SHUV-positive maternal epithelial cells were detected throughout the placentomes, with occasional detection of infected trophoblasts (Figure 1h). Interestingly, inoculation of pregnant ewes with AKAV at gestation day (gd) 32 revealed infection of trophoblasts at 5 dpi, whereas infected maternal epithelial cells were not detected [27].

The absence of viremia in one ewe, the low-level viremia in two ewes, and the relatively late onset of viremia in ewe #197, may suggest that the SHUV isolate used in the present work, being originally isolated from a cow, is not optimally adapted to sheep. The virus that was successfully isolated from the plasma of sheep #197 may, therefore, be more suitable for future experimental inoculation of the same species. 

### 3.2. SHUV Targets Human Trophoblasts

**Figure 2:**
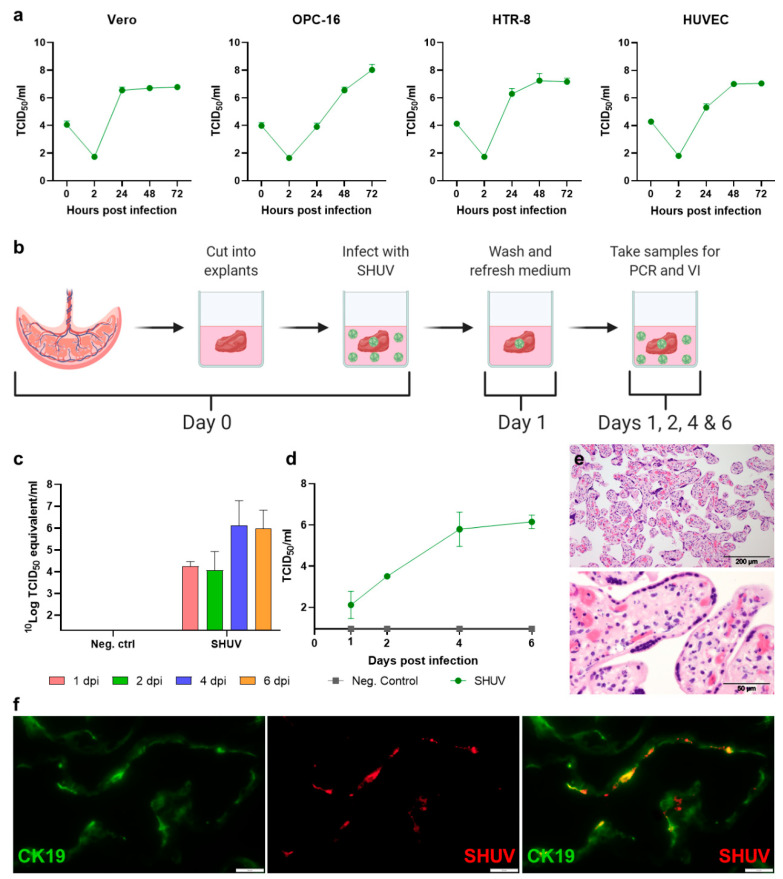
Replication of SHUV in human placental cell lines and explants. (**a**) Growth kinetics of SHUV in Vero cells, ovine placental cells (OPC-16), human trophoblast cells (HTR-8), and human umbilical vein endothelial cells (HUVEC). Cells were incubated with a multiplicity of infection (MOI) of 0.01, and samples were taken at 0, 2, 24, 48, and 72 hours post-infection. (**b**) Procedure used to evaluate replication of SHUV in human placental explants. On day 0, explants (n = 4 per timepoint) were incubated with SHUV. At 1 day post-infection, medium was removed, and explants were washed 3 times with PBS. Explants and supernatant samples collected at 1, 2, 4, and 6 dpi were used for RNA isolation and virus titrations. (**c**) Viral RNA detected in organ suspensions of placental explants. (**d**) Virus titers detected in pooled supernatant samples collected during two separate experiments. (**e**) HE staining of normal human placental explants at 4 dpi showing the fetal villi. (**f**) Detection of SHUV-infected syncytiotrophoblasts by immunofluorescence at 4 dpi. Syncytiotrophoblasts were visualized using an antibody specific for cytokeratin-19 (CK19). SHUV antigen was detected using an antibody recognizing the SHUV Gc ectodomain. White bars: 20 µm. Data points are means with SD.

To investigate if SHUV can infect cells of the human placenta, a human trophoblast cell line (HTR-8), and a human endothelial cell line, derived from an umbilical vein (HUVEC cells), were incubated with the virus. Vero E6 cells and an ovine placental cell line were taken along as positive controls. SHUV efficiently replicates in all these cell lines (Figure 2a).

After demonstrating that SHUV can replicate in both HTR-8 and HUVEC cells, explants from human term placentas, obtained from healthy donors after caesarean section, were incubated with SHUV (Figure 2b). On days 1, 2, 4, and 6 dpi, placental explants were used for RNA isolation (n = 4 per placenta). At the same timepoints, supernatants were also collected for virus titration. RT-PCR on RNA extracted from the explants revealed that after removal of the inoculum, 10^4.5^ TCID_50_ equivalents/mL viral RNA was detected (Figure 2c). Viral RNA levels did not increase before 2 dpi; however, a 2 log_10_ increase was observed between days 2 and 4, which was maintained until day 6. Titration of the supernatants revealed a steady increase of infectious SHUV from day 1 onwards to reach a final titer of 10^6^ TCID_50_/mL (Figure 2d). These results demonstrate that human placental cells are susceptible and permissive to SHUV.

Immunohistochemical analysis of the human placental explants subsequently revealed SHUV antigen distributed throughout the placental explants. SHUV-specific staining was restricted to syncytiotrophoblasts surrounding the fetal villi, inferring that SHUV can infect this cell type (Figure 2e,f). The finding that human syncytiotrophoblasts are susceptible and permissive to SHUV replication is notable, as these cells form the only continuous barrier between the maternal and fetal circulation and are generally highly resistant to virus infection. 

In conclusion, our results demonstrate the ability of SHUV to replicate in human placental trophoblasts. Although evidence of vertical transmission in humans is currently lacking, our findings call for increased awareness of SHUV infections in humans.

## Data Availability

The data presented in this study are available on request from the corresponding author.

## Supplementary Materials

The following are available online at https://www.mdpi.com/2076-0817/10/1/17/s1, Figure S1: Standard curve to calculate TCID50 equivalents of SHUV.
