# Supplementary material for: Shuni Virus Replicates at the Maternal-Fetal Interface of the Ovine and Human Placenta
Source: Pathogens. 2020 Dec 29;10(1):17. doi: 10.3390/pathogens10010017 (PMC7823754; doi:10.3390/pathogens10010017)
Supplement: Supplementary file 1 [file pathogens-10-00017-s001.pdf]

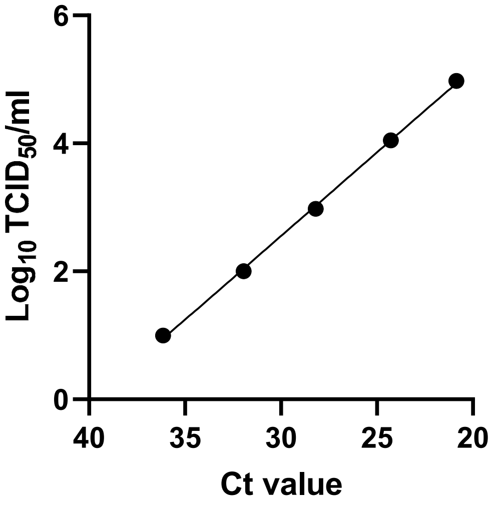

Supplementary Figure S1. Standard curve to calculate $TCID_{50}$ equivalents of SHUV. SHUV RNA of a stock of known $TCID_{50}$ titer was lysed in Trizol, followed by isolation of RNA using the Direct-zol RNA miniprep kit (Zymo Research) according to the manufacturer's instructions. A 10-fold dilution series was prepared in water, and aliquoted for RT-qPCR runs.
